# Transcatheter Mitral Valve Replacement Using Transcatheter Aortic Valve or Dedicated Devices: Current Evidence and Future Prospects

**DOI:** 10.3390/jcm12216712

**Published:** 2023-10-24

**Authors:** Victor Quentin, Jules Mesnier, Clémence Delhomme, Neila Sayah, Paul Guedeney, Olivier Barthélémy, Gaspard Suc, Jean-Philippe Collet

**Affiliations:** 1 Department of Cardiology, Hôpital Bichat, Assistance Publique-Hôpitaux de Paris, Paris Cité Université, 75005 Paris, France; 2 ACTION Study Group, INSERM UMRS_1166, Institut de Cardiologie (AP-HP), Sorbonne Université, 75013 Paris, France

**Keywords:** transcatheter mitral valve replacement, mitral regurgitation

## Abstract

Transcatheter mitral valve replacement (TMVR) is a novel and evolving field dedicated to addressing the therapeutic challenges posed by patients at high surgical risk with mitral valve disease. TMVR can be categorized into two distinct fields based on the type of device and its specific indications: TMVR with transcatheter aortic valves (TAV) and TMVR with dedicated devices. Similar to aortic stenosis, TMVR with TAV requires a rigid support structure to secure the valve in place. As a result, it is indicated for patients with failing bioprothesis or surgical rings or mitral valve disease associated with severe mitral annular calcification (MAC), which furnishes the necessary foundation for valve anchoring. While TMVR with TAV has shown promising outcomes in valve-in-valve procedures, its effectiveness remains more contentious in valve-in-ring or valve-in-MAC procedures. Conversely, TMVR with dedicated devices seeks to address native mitral regurgitation, whether accompanied by MAC or not, providing an alternative to Transcatheter Edge-to-Edge Repair (TEER) when TEER is not feasible or expected to yield unsatisfactory results. This emerging field is gradually surmounting technical challenges, including anchoring a valve in a non-calcified annulus and transitioning from the transapical route to the transeptal approach. Numerous devices are presently undergoing clinical trials. This review aims to furnish an overview of the supporting evidence for TMVR using TAV in each specific indication (valve-in-valve, valve-in-ring, valve-in-MAC). Subsequently, we will discuss the anticipated benefits of TMVR with dedicated devices over TEER, summarize the characteristics and clinical results of TMVR systems currently under investigation, and outline future prospects in this field.

## 1. Introduction

Transcatheter mitral valve replacement (TMVR) has been developed to treat patients with high or prohibitive surgical risk using either transcatheter valves for failed surgical annuloplasty, bioprothesis, or severe mitral annular calcification (MAC) or dedicated devices for severe mitral valvular regurgitation (MR). Severe MR is one of the most prevalent valvular conditions in Western countries, primarily due to its strong association with age, and it is further linked to adverse cardiac outcomes, including the development of heart failure [1,2,3,4]. Surgical intervention remains the standard of care according to international guidelines but is not feasible in all patients in the oldest patients. Consequently, at least 30% of patients with severe mitral disease are left untreated, with grim consequences for both their functional status and prognosis [4,5]. To address this issue, new interventional approaches have been developed in the last few years, specifically transcatheter edge-to-edge repair (TEER) and TMVR with dedicated devices. Substantial evidence and experience have accumulated with TEER for both functional and organic severe MR, making it the primary alternative to surgical management [4,5]. However, some of these patients are ineligible for TEER due to challenging anatomy or are poor candidates because of high risk of residual MR, which is associated with poor outcomes [6,7]. As such, there is a growing interest in dedicated TMVR devices to overcome the limitations of TEER. In this review, we will first examine the results of TMVR with the balloon-expandable valves designed for transcatheter aortic valve replacement. Next, we will focus on dedicated TMVR devices, highlighting their potential benefits compared to TEER, and describing the dedicated devices under clinical investigation and their available associated evidence. Pre-procedural planning and procedural characteristics will only be briefly addressed in this review.

## 2. TMVR with Transcatheter Aortic Valve

The first-in-man implantation of a balloon-expandable transcatheter aortic valve (TAV) device in a failing mitral bioprothesis occurred in 2009 using a transapical approach [8]. Subsequently, TMVR with TAV for failing surgical rings or severe MAC was realized in 2011 and 2013, respectively, delineating the three specific clinical situations in which TMVR with TAV can be performed: Valve-In-Valve (ViV), Valve-In-MAC (ViMAC), Valve-In-Ring (ViR) [9,10]. Since then, the field of TMVR with TAV has evolved significantly. The number of TMVR with TAV procedures has been steadily increasing, with 1120 procedures performed in 2019 in the United States of America. Among these, 78.0% were ViV TMVR, 11.7% were ViR TMVR, and 10.3% were ViMAC TMVR [11]. This development underscores the technical feasibility of such procedures and the unaddressed demand for a less invasive alternative to surgery in patients with severe MR or stenosis with these conditions. In the case of failed surgical prothesis, degeneration is a natural occurrence expected between 10 and 15 years after the initial surgery. Additionally, a portion of patients who have undergone mitral valve repair will eventually require a new surgical intervention [2,7,12]. However, the period between the initial surgery and the potential need for a redo has seen patients aging and developing more comorbidities, significantly increasing the surgical risk—also increased by the redo procedure. In fact, redo mitral surgery in these specific indications is associated with a mortality between 10% to 20% [13]. As a result, TMVR with TAV has gained attention in recent years, particularly after reports demonstrated favorable clinical and echocardiographic outcomes after 1 year follow-up [14]. MAC is a degenerative disease that can lead to significant mitral regurgitation, stenosis, or both. While its pathophysiology and prevalence remain poorly understood due to the absence of a standardized definition, MAC is associated with aging, comorbidities, and unfavorable cardiovascular outcomes, including increased all-cause mortality [15,16,17]. Surgical management is a technical challenge, and TMVR with TAV has emerged as an attractive solution for these high-risk patients [18].

### 2.1. Screening and Procedural Technique of TMVR with TAV

Like any percutaneous technique, patient selection for TMVR is critical and requires a comprehensive assessment, including a cardiac gated computed tomography (CT) scan and an ultrasound evaluation, to confirm technical feasibility and rule out potential contraindications (such as infective endocarditis, prosthesis, or ring disinsertion, paravalvular leaks, or valve thrombosis) [19]. Dimensions of the mitral annulus to size the TAV device and assess the risk of left ventricle outflow tract obstruction (LVOT) are key measurements. Neo-LVOT is an important predictive factor for TMVR results and a common reason for screening failure [20]. In the case of MAC, a sufficient degree and circularity of calcification is necessary to anchor the prosthesis, and the size of the native mitral annulus should fall within the range covered by the Sapien valve to prevent valve migration [21]. Furthermore, the severity and extent of calcifications strongly influence the results of TMVR and should, therefore, be thoroughly evaluated [19,22,23]. In ViV-TMVR, the true internal diameter should be meticulously assessed, relying on both the size measured on the CT-scan and the internal diameter provided by the manufacturer. For ViR-TMVR, the anatomy, shape, and rigidity of the surgical ring will predict the risk of paravalvular leak after the procedure, especially in rigid rings that preclude perfect circularization of the valve [24].

TMVR procedures are typically conducted under general anesthesia and guided by transesophageal echocardiography (TEE) and fluoroscopy for optimal valve positioning, especially in ViMAC cases, where the annulus plane may not be clearly determined by fluoroscopy. Historically, the transapical route was more common due to its technical simplicity and previous experience with transcatheter aortic valve procedures [11]. However, due to its invasiveness and less favorable results in the field of transcatheter aortic valve replacement, the less invasive transseptal route has taken over [19]. Finally, in rare cases, a surgical transatrial route may be employed, necessitating extracorporeal circulation for patients with complex MAC and a high risk of left ventricular outflow tract obstruction [19].

### 2.2. Outcomes of TMVR with TAV

In a systematic review published in 2021, results from studies, each including at least 20 patients, were pooled together to analyze outcomes in the three indications, mixing indications for severe MR or stenosis [19]. ViV TMVR exhibited the most favorable outcomes, with a procedural success rate exceeding 90%, compared to approximately 80% in ViR TMVR and around 70% in ViMAC TMVR. Similarly, the most favorable 30-day outcomes were observed in the ViV TMVR, with the lowest rates of death, stroke, LVOT obstruction, and valve embolization, and at 1-year follow-up with cardiovascular mortality of 12% [19]. Results were notably less favorable in the ViMAC population, with a 30-day mortality of approximately 20% reaching nearly 50% at the 1-year follow-up. It was also the group with the highest rate of stroke, LVOT obstruction, and valve embolization. It is essential to note that significant variations across observational studies existed, emphasizing the need for standardized definitions in trials and registries. Additionally, results beyond the one-year follow-up were limited.

Subsequently, the MITRAL (Mitral Implantation of Transcatheter Valves) trial, a prospective study assessing the outcomes of TVMR with TAV using the Sapien XT and Sapien 3 valves, provided similar results when compared to the pooled analysis of Urena et al. [25,26,27,28]. This observational trial adopted a 3 single-arms design (ViV, ViR, ViMAC) across 13 different United States medical centers and included 91 patients overall. The access route was transseptal for all ViV and ViR TMVR procedures, while transatrial and transapical routes were reported in half of the ViMAC cases. The technical success rate defined as mean transvalvular gradient <10 mmHg and severity of residual MR < grade 2 neared 80% overall with notable differences depending on the indication: 100% in ViV, 66.7% in ViR, and 74.2% in ViMAC groups. All-cause mortality at 30 days was 3.3% in ViV, 6.7% in ViR, and 16.7% in ViMAC groups, illustrating the technical complexity and weight of associated comorbidities in this last group. Notably, screening failure varied significantly across the different indications, with the highest rate observed in MAC patients, where approximately 66% of screen failure was reported, compared to 16.5% in the surgical ring group and 21% in the ViV group. One-year survival was 97% in ViV group, whereas it was considerably lower in ViR group and ViMAC group (77% and 65%, respectively). As expected, the transeptal approach presented lower mortality rates at 30 days and 1 year compared to alternatives in the ViMAC group (6.7% and 26.7% for transeptal vs. 21.4% and 38.5% for the transatrial route, 100% at 30 days for transapical) [27]. At 2-year follow-up, successful TMVR led to persistent functional improvement compared to baseline in survivors [29]. However, while mortality rate remained stable in ViV and ViMAC groups (6.7% and 39.3%, respectively), it increased to 50% in the ViR group. This may be attributed to the rate of residual MR and lower initial LVEF in this group. Nevertheless, it provides a clear indication of unfavorable early outcomes following ViR TMVR. Importantly, a majority of deaths involving the ViMAC group at 2-year follow-up occurred between implantation and the following year, suggesting that a subgroup of MAC patients can indeed benefit from the procedure [29]. Final results after 5-years follow-up confirmed ViV patients showed the lowest rates of death (21.4% in ViV group, vs. 65.5% in ViMAC group and 67.9% in ViR group) but also mitral valve reintervention and hemolytic anemia compared than the other two groups [30].

### 2.3. Perspectives of TMVR with TAVR

Overall, TMVR with TAV has demonstrated excellent procedural and mid-term outcomes in ViV TMVR patients while mitigated in ViR patients, raising the futility of such a procedure when TEER can be performed in selected patients within this population [19]. For MAC patients, both procedural and short-term outcomes are less favorable, with one-third of patients dying within the first year. Consequently, ViR and ViMAC TMVR candidates should undergo thorough evaluation by an experienced heart team, and the associated risks should be carefully shared with the potential candidates. More detailed analysis and further trials are required to identify the most suitable candidates for such intervention. Finally, these mitigated results highlight the need for alternative solutions in ViR and ViMAC patients, such as TMVR with dedicated devices.

## 3. TMVR with Dedicated Devices

### 3.1. TMVR Potential Advantages Compared to TEER

**Figure 1:**
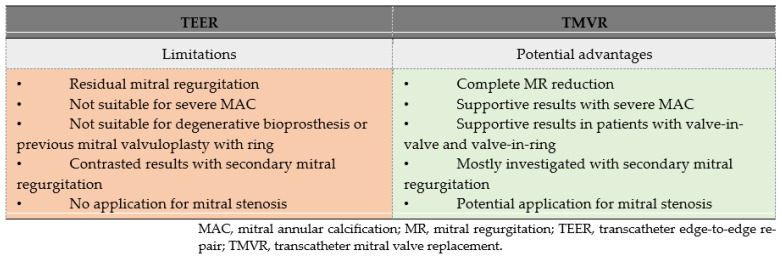
Potential Limitations of TEER Addressed by TMVR.

The potential benefits of TMVR compared to TEER limitations are summarized in Figure 1. TEER serves as the first-line percutaneous interventional alternative to surgery for managing symptomatic MR [31,32,33]. It is established as a safe and effective procedure, delivering sustained results over time [33,34] but with remaining pending challenges. First, post-procedural MR persists in approximately one-third of TEER recipients, allowing for the potential progression of MR in the future, a factor associated with unfavorable outcomes [33,35,36]. Second, the outcomes of TEER in patients with secondary MR, particularly those with severely altered LVEF, produced mixed results in randomized trials [37,38]. Finally, a significant proportion of patients did not qualify for TEER due to anatomical constraints, such as calcified leaflets or short posterior mitral leaflets with limited mobility. In fact, nearly 25% of patients with secondary MR in the MITRA-FR trial did not meet the anatomical eligibility criteria [37]. Similarly, patients with severe MAC were excluded from randomized trials, and although recent observational data suggest the feasibility of TEER in a limited number of selected patients, further investigations are warranted [39].

TMVR has the potential to address some of the existing limitations associated with TEER. TMVR will likely have less residual MR, might effectively manage secondary MR, and provide an interventional option for patients whose mitral anatomy is unsuitable for TEER [40]. Several studies have explored whether TMVR can indeed overcome the limitations of TEER. In a recent retrospective cohort comparing both technics in patients with impaired LVEF (mean 40% for TMVR and 41% for TEER), regardless of MR etiology, TMVR demonstrated the ability to induce positive LV remodeling, notably reducing the LV end-systolic volume index, an effect not observed in the TEER group [41]. In the CHOICE-MI registry, 262 patients who underwent TMVR were matched with patients treated with TEER for severe secondary MR management. There was no difference in mortality rates within 30 days or 1 year. However, TMVR recipient exhibited a significantly greater reduction of MR and experienced superior symptomatic improvement [42]. Nonetheless, it is essential to interpret these supportive results with caution, and further investigation is warranted through dedicated trials.

### 3.2. Screening and Procedural Technique of TMVR with Dedicated Devices

Corelab comprehensive echocardiographic and gated CT-scan are mandatory [43,44]. Such evaluation encompasses a thorough analysis of the mitral valve and its apparatus, including the annulus shape and size, anterior leaflet length, chordae tendineae locations, papillary muscle locations, left ventricular and atrial dimensions, left ventricular outflow tract (LVOT) and anticipated neo-LVOT, aorto-mitral angle, and upper septal thickness [44,45,46]. It is important to note that specific anatomical characteristics will vary depending on the individual device, its anchoring mechanism, and the chosen delivery route.

While most devices are primarily designed for transapical access, which facilitates the implantation of a large device matching the dimensions of the native mitral valve and ensures optimal alignment, there is a growing interest in less invasive approaches. Most devices are designed for transapical access, facilitating the implantation of a substantial device matching the large dimensions of the native mitral valve and ensuring optimal alignment. Consequently, many devices have already incorporated transseptal delivery systems. Some devices, however, may struggle to overcome the technical challenge of transitioning toward transfemoral access [47,48,49].

### 3.3. Dedicated Devices for TMVR: Description, Outcomes, and Ongoing Trials

**Figure 2:**
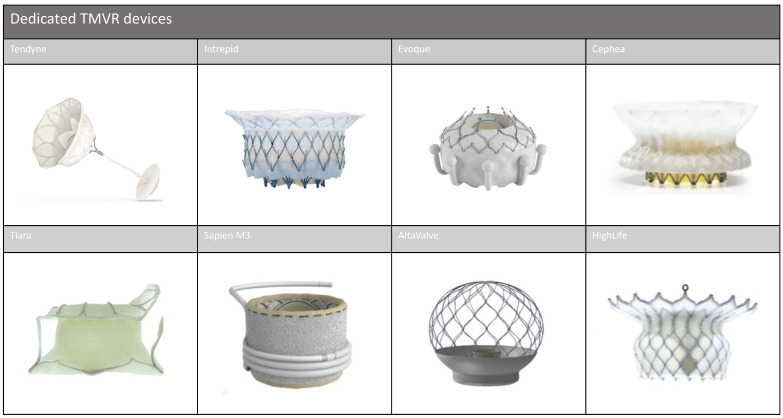
Dedicated TMVR Devices. TMVR: transcatheter mitral valve replacement.

Current data on dedicated devices are limited, as most of them are still in the early stages of development. In this section, we provide an overview of these devices, which are illustrated in Figure 2, and outline ongoing trials, which are detailed in Table 1.

#### 3.3.1. Tendyne (Abbott, Menlo Park, California)

Description: The Tendyne prosthetic device is made of a trileaflet porcine pericardial valve supported by two self-expending nitinol frames: an inner and an outer frame. The outer frame is designed to conform to the patient’s native mitral annulus. A high-molecular-weight polyethylene tether, connected to a pad positioned over the transapical access site, secures the device to the ventricular apex. This configuration allows for device repositioning and retrieval [47,50]. The Tendyne device is delivered through transapical access, with a 34-F delivery sheath.

Available data: Two studies have reported prospective data on the Tendyne device: the early feasibility cohort that included 100 patients and a second that enrolled nine patients with severe MAC. The technical success rate was 96%. Mortality was 6% at 30-day and 27.6% at 1-year follow-up. The most common major adverse event was major or life-threatening bleeding, occurring in 19% of cases at 30 days. Freedom from MR was excellent in patients with successful device implantation, with only one moderate MR (1.1%) at hospital discharge. At 1 year, 98.4% of survivors had no MR or trivial MR, along with sustained functional improvement. At the 2-year follow-up, mortality was 39%, and 93.2% of survivors had no MR, alongside persistent symptom improvement [51].

Ongoing trials: In addition to the results from the extended follow-up of the early feasibility study, three ongoing trials are currently underway. The SUMMIT trial (Clinical Trial to Evaluate the Safety and Effectiveness of Using the Tendyne Transcatheter Mitral Valve System for the Treatment of Symptomatic Mitral Regurgitation—NCT03433274) aims to compare the Tendyne device with MitraClip in patients with moderate-to-severe or severe MR due to severe MAC. The Tendyne RESOLVE-MR study (Real World Study of the Tendyne Mitral Valve System to Treat Mitral Regurgitation—NCT04818502) is an ongoing European post-market registry designed to provide real-life data. Lastly, a single-arm study is assessing feasibility in patients with severe MAC, with completion expected in 2024 (NCT03539458).

#### 3.3.2. Tiara (Neovasc Inc., Richmond, BC, Canada)

Description: The Tiara device features a central structure composed of a bovine pericardial valve supported by a self-expanding nitinol platform. This platform is saddle-shaped to conform to the native mitral valve anatomy. The entire support structure is covered by a skirt, with the notable feature of asymmetry on its atrial side, allowing for optimal seating of the prosthesis. On the ventricular side, anchoring reliefs are present, including an anterior relief oriented towards the mitroaortic trigone and two posterior reliefs. The Tiara device is exclusively implanted via the transapical approach [48]. The delivery system for the Tiara device consists of a 36-F system for the 35 mm valve and a 40-F system for larger valves.

Available data: Currently, there is limited available data on the use of the Tiara valve. Initial evaluations involving 50 patients report an implantation success rate of 95%, with a 30-day mortality rate of 8.5% [52].

Ongoing trials: Additional data on the Tiara device will be provided by two ongoing trials: the international TIARA-I (Early Feasibility Study of the Neovasc Tiara™ Mitral Valve System—NCT02276547) and the TIARA-II CE MarkTrial (Tiara Transcatheter Mitral Valve Replacement Study—NCT03039855). Additionally, there is an ongoing engineering process for a transseptal delivery system [53].

#### 3.3.3. Intrepid (Medtronic, Minneapolis, Minnesota)

Description: The Intrepid bioprosthesis is based on a trileaflet bovine pericardial valve encased in a nitinol frame. This self-expanding frame includes a circular inner stent surrounding the valve and a larger outer stent pressed onto the mitral annulus to ensure secure fixation. Both stents are covered by a polyester fabric skirt. The outer frame is designed to accommodate shape variations during the cardiac cycle, while the inner frame maintains a circular shape of the valve. Anchoring and sealing rely on the oversizing of the outer frame and external cleats that act as frictional members to engage native leaflets. There is a gap between the two stents to promote tissue colonization to further anchor the system [54]. Initially, the Intrepid device was available only for a transapical approach, but a 36-F transseptal delivery system has since been developed [55].

Available data: Results from the early feasibility trial using the transapical device have already been reported and demonstrated successful implantation in 48/50 selected patients (96%) [56]. The 30-day mortality rate was 14% (7/50). All survivors who received the Intrepid valve had either mild or no residual MR at the 30-day echocardiogram evaluation and clinical improvement (defined as class I or II of the New York Heart Association classification) was observed in 79% of cases [56]. The transseptal version was evaluated in a single-arm non-randomized prospective study involving 15 patients and yielded promising results, with a success rate of 93%. All patients were alive at 30 days. However, there was 6 (40%) access site bleeding. All patients with successful implantation had trace or no valvular or paravalvular MR [57].

Ongoing trials: The APPOLLO trial (Transcatheter Mitral Valve Replacement with the Medtronic Intrepid TMVR System in Patients with Severe Symptomatic Mitral Regurgitation—NCT03242642) is a large ongoing multicenter, prospective, non-randomized trial, with an estimated enrollment of 1350 patients. This trial aims to assess the efficacy of the Intrepid valve in patients with moderate-to-severe or severe symptomatic MR who are ineligible for transcatheter edge-to-edge repair (TEER), with or without mitral annular calcification (MAC). Patients will be divided into two cohorts based on the presence of MAC [55]. Additionally, the APPOLLO-EU study (NCT05496998) is currently recruiting up to 360 patients in Europe to treat severe MR using the transfemoral Intrepid system.

#### 3.3.4. AltaValve (4C Medical Technologies, Minneapolis, Minnesota)

Description: The AltaValve stands out with its unique design, positioned exclusively on the atrial side of the mitral valve, making it supra-annular. This approach is intended to reduce the likelihood of LVOT obstruction, valve embolization, and interference with sub-valvular chordae. It features a spherical structure securely attached to the atrial walls, housing a trifoliate valve composed of bovine tissue at its base. The skirt of the device covers the lower part towards the valve to prevent peri-valvular leaks. Initially, the device was delivered using a 32-F sheath via transapical access and was repositionable and partially retrievable. Subsequently, a 29-F transseptal delivery system has been developed [58,59].

Available data: Data on the AltaValve are limited to case reports. The overall implantation success rate was 100%, and durable results were observed at 6 months for both access routes [59,60].

Ongoing trial: An early feasibility study is currently enrolling patients (NCT03997305).

#### 3.3.5. Sapien M3 (Edwards Lifesciences, Irvine, California)

Description: The Sapien M3 valve consists of two distinct parts deployed sequentially: the dock and the valve. The dock is constructed from nitinol and covered with polytetrafluoroethylene, designed to encircle the chordae of the sub-valvular apparatus to provide anchoring for the valve. The valve itself is identical to the 29-mm Sapien 3, originally designed for percutaneous aortic replacement, with the addition of a knitted external polyethylene terephthalate membrane on the outer face of the valve. The Sapien M3 can be implanted through a 20-F femoral introducer, and the dock remains fully recapturable until final detachment with the delivery catheter [61].

Available data: In the first-in-human experience, technical success with the Sapien M3 device was achieved in 9 of 10 patients. At 30 days, all patients were alive, and all implanted valves were functional without MR and without an increase in mean transmitral gradients [62]. Preliminary results from the ongoing early feasibility study were presented, indicating a successful implantation rate of 88.6% in the first 35 patients. At the 30-day follow-up, one patient (2.9%) had died, and 8.6% had experienced a stroke. MR was significantly reduced, with 87.9% of patients exhibiting no or trace residual MR [63].

Ongoing trial: The ENCIRCLE trial (SAPIEN M3 System Transcatheter Mitral Valve Replacement Via Transseptal Access—NCT04153292) is a prospective single-arm, multicenter study aimed at establishing the safety and effectiveness of the Sapien M3 valve in subjects with symptomatic moderate to severe or severe MR, deemed unsuitable for surgical or other transcatheter options.

#### 3.3.6. EVOQUE Mitral System (Edwards Lifesciences)

Description: The EVOQUE valve comprises a trileaflet valve within a self-expanding nitinol frame. It utilizes a specific anchoring system that captures the mitral leaflets and subvalvular chordae using hooks. Once in the left ventricle, the device is progressively deployed, exposing the anchors, which retract onto the native mitral leaflets and chordae as the device is further deployed. Additionally, the atrial portion of the device features a sealing skirt. The EVOQUE valve is delivered through a transfemoral approach with a 28-F sheath [64].

Available data: Initial results with the EVOQUE valve in 14 North American patients were published [64]. Technical success was achieved in 13 patients (93%), with one conversion to open surgery. At the 30-day follow-up, the survival rate was 93%, and the rates of stroke and major or life-threatening bleeding were 14.3% and 21.4%, respectively.

Ongoing trial: The MISCEND trial is an ongoing study evaluating the safety and performance of the EVOQUE mitral valve at 30 days (NCT02718001).

#### 3.3.7. CEPHEA (Abbott, Menlo Park, California)

Description: The CEPHEA valve is a bovine trileaflet valve incorporated into a double-disc structure made of self-expanding nitinol, shaped like a vase. The prosthesis is anchored by the axial force exerted on the mitral annulus. Due to the self-expanding nature of the external disk, the CEPHEA valve can adapt to various anatomies while isolating the valve from external deformation. The valve is delivered via a transfemoral approach [65].

Available data: A total of four cases with the CEPHEA valve have been reported. Technical success was achieved in all cases, with no mortality, no stroke, no significant bleeding, and no residual MR at the 30-day evaluation. Functional status also improved in all patients [65,66].

Ongoing trial: An early feasibility study is ongoing in North America (NCT05061004).

#### 3.3.8. HIGHLIFE (Highlife SAS, Irvine, California)

Description: The Highlife prosthesis features a two-part design consisting of an anchoring ring and a valve. The ring is placed around the mitral subannular apparatus to create a rigid support for anchoring the valve, which has a single definitive size of 31 mm. The implant is delivered retrogradely through the aortic valve using an 18-F catheter delivery system. The valve itself consists of a nitinol self-expanding frame and was initially delivered via a 39-F transapical access [67,68]. A transfemoral delivery system has since been developed [69].

Available data: Data are available on the first 15 patients treated with the transapical system, while two reports (the first-in-human and a 4 patients’ study) were reported with the transfemoral device. With the transapical system, 3/15 patients had died at 30 days [68]. With the transfemoral system, technical success was achieved in all 4 patients with excellent valvular function, and all patients were alive at 30 days [70].

Ongoing trial: Several single-arm studies are underway in France, Australia, China, and the United States (NCT02974881, NCT04029337, NCT04029363, NCT05610566).

#### 3.3.9. Pooled Results of TMVR with Dedicated Devices

A recent meta-analysis, comprising data from 12 studies involving 347 patients who underwent transcatheter implantation of dedicated TMVR devices, demonstrates promising outcomes. TMVR was found to significantly reduce severe mitral regurgitation (MR) and improve functional status. The pooled technical success rate was 95.4%, with only 0.6% of patients experiencing moderate to severe or severe MR at the 30-day mark, even though the majority of TMVR procedures were performed in patients with secondary MR (58%) [71]. There was a notable improvement in the functional status of patients following the procedure. However, a major limitation of TMVR was the occurrence of major bleeding occurring in 15.6% of cases at 30 days.

### 3.4. Perspectives on TMVR with Dedicated Devices

While TMVR is making significant progress, there remain opportunities for improvement and a need for more robust evidence.

One of the key theoretical advantages of dedicated TMVR systems is their ability to address a wide range of anatomies, particularly those unsuitable for TEER. However, it is worth noting that patients included in first-in-human or early feasibility studies, which constitute the majority of the current data on dedicated TMVR devices, were carefully selected by the manufacturing companies and scientific boards. Unfortunately, the exact number of patients ultimately deemed ineligible for TMVR is unknown, as many studies did not provide data on screen failure. Screen failure in TMVR can result from various factors, including out-of-range mitral annulus size (as most devices have a limited therapeutic range), the risk of LVOT obstruction, or inappropriate anatomy. Presently, we are in a position where we carefully select patients for a specific device rather than the other way around.

Addressing technical challenges has not allowed for rapid development of dedicated devices. One significant challenge is transitioning from the transapical to the transfemoral approach, which is likely to become the default strategy in the future once these devices are commercially available. While technical success has been reported with the transapical route in TMVR, it has been associated with a slight increase in procedural mortality compared to the transfemoral approach [72]. Moreover, the detrimental effects of the transapical approach in the transcatheter aortic valve replacement (TAVR) field are well-documented, and it is highly probable that similar concerns exist in TMVR recipients. The engineering challenge of designing a low-profile, flexible delivery sheath that can be manipulated safely is complex but crucial for patient safety. Reducing the size of the delivery system is another challenge that must be addressed to enhance procedural safety.

Lastly, there is a strong anticipation for randomized trials, large registry, and long-term outcomes. Key areas of interest include valve durability, the incidence of valve thrombosis, late valve embolization, and the management of antithrombotic therapy. Comprehensive long-term data will be instrumental in solidifying the role of TMVR in the treatment landscape for mitral valve disease.

## 4. Conclusions

**Figure 3:**
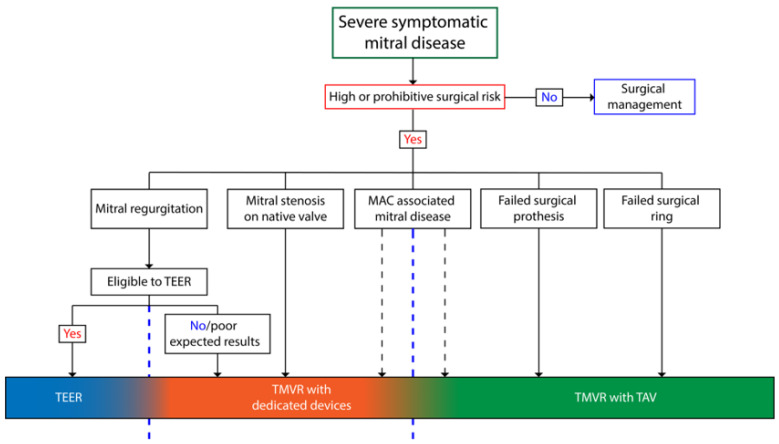
Algorithm of Available Therapies According to Mitral Valve Disease.

TMVR has emerged as a promising approach for the treatment of mitral valve disease, offering an interventional alternative for previously untreated patients (Figure 3). Transcatheter aortic valve replacement with dedicated valves is the primary alternative to repeat surgical interventions for failed bioprostheses, while further investigations are needed to identify the specific patients with MAC or failing rings who can benefit from the procedure. TMVR with dedicated devices represents a novel strategy for addressing mitral valve disease, aiming to overcome some of the limitations associated with TEER, particularly in high surgical risk patients. The challenges related to the intricate anatomy of the mitral valve and apparatus, notably in achieving effective valve anchoring and adapting to transfemoral access, have presented hurdles to the widespread development of dedicated TMVR devices. Nonetheless, several devices have shown promising early results in carefully selected patients and are currently undergoing clinical investigation. The results of forthcoming trials hold the promise of delivering robust clinical evidence in this rapidly evolving field.

## 5. Key Points

- There is an unmet clinical need for definitive treatment options for patients with mitral valve disease who are ineligible for surgery or TEER. TMVR represents a pioneering interventional solution for this underserved patient population.
- TMVR with TAV has demonstrated its safety and effectiveness, particularly in patients with failing mitral bioprostheses. However, outcomes have been less favorable in patients with MAC or those with previously failed surgical repairs. Therefore, a meticulous evaluation of patients by experienced heart teams should be considered a prerequisite before embarking on ViMAC and ViR procedures.
- The ongoing development of dedicated prosthetic devices shows promise, with initial results indicating their potential. The eagerly anticipated results of future trials will be instrumental in shaping the landscape of this expanding field.

## Figures and Tables

**Table 1:** Ongoing Trials for Each Dedicated TMVR Devices.

Device	Trial/Study	Method	Inclusion Criteria	Access	N. of Patient Expected	Primary Outcome	Expected Date of First Results
Tendyne	SUMMIT trial—NCT03433274	Prospective, controlled, multicenter clinical trial with 4 arms: - 1:1 randomization between Tendyne and Mitra-Clip cohorts - Patients non-eligible for TEER (nonrepairable arm) - Severe MAC arm - Severe MAC continued access	- LVEF >30% - MR grade ≥ 3+ - And/or nonrepairable MR or severe MAC	TA	n = 958	Survival free of heart failure hospitalization at 12 months post index procedure	June 2024
	Feasibility Study of the Tendyne Mitral Valve System in Mitral Annular Calcification—NCT03539458	Prospective, single-arm, multicenter in eligible subjects with symptomatic, severe mitral regurgitation and severe MAC	- Symptomatic severe MR - Severe MAC	TA	n = 30	Composite endpoint of device success and freedom from device or procedure-related serious adverse events at 30 days	October 2024
	RESOLVE-MR study—NCT04818502	Single-arm, multicenter study to support post-market follow-up requirements of CE Mark	-	TA	n = 200	MR elimination at 1 year: free from surgical removal/replacement or transcatheter mitral valve implantation and MR < grade I	May 2025
Tiara	TIARA-I trial—NCT02276547	Single-arm, multicenter study	- Severe MR - High surgical risk - NYHA III or IV	TA	n = 27	Freedom from all-cause mortality and major adverse events from the time of implant procedure to 30 days, or hospital discharge	February 2025
	TIARA-II CE Mark Trial—NCT03039855	Single-arm, multicenter study	- Severe MR - High surgical risk - NYHA III or IV	TA	n = 115	Freedom from all-cause mortality, major adverse events, reduction of MR to optimal or acceptable at 30 days	January 2026
Intrepid	Early Feasibility Study of the TMVR Transseptal System—NCT02322840	Prospective, multicenter, non-randomized trial	- Symptomatic MR ≥ 3+	TA/TS	n = 33	Adverse events associated with the delivery and/or implantation of the device.	Completed, follow-up until 2028
	APOLLO Trial—NCT03242642	Multicenter, global, prospective, non-randomized, interventional, pre-market trial. Two cohorts: - without severe MAC - with severe MAC	- Symptomatic MR ≥ 3+ or MR grade 3 and mitral stenosis in the MAC cohort - Ineligible for TEER - Inoperable	TATS	n = 1350	All-cause mortality and heart failure hospitalization in 1-year composite	October 2026
	APOLLO-EU Trial—NCT05496998	Prospective, single-arm, multicenter, interventional, pre-market trial	- MR ≥ 3+ - Ineligible for TEER - Inoperable	TS	n = 360	Safety: all-cause mortality at 1-year. Efficacy: Percentage of subjects with none/trace or mild MR at 30 days	November 2026
AltaValve	AltaValve Early Feasibility Study—NCT03997305	Prospective, single-arm, multicenter study	- NYHA ≥ 2 - Severe MR - LVEF ≥ 30% - High surgical risk	N/A	n = 15	Cardiac death, stroke, mitral valve-related repeated intervention at 30 days	September 2025
Sapien M3	Early Feasibility Study of the SAPIEN M3—NCT03230747	Prospective, single-arm, multicenter early feasibility study	- MR ≥ 3+ - NYHA ≥ 2 - High surgical risk	TS	n = 74	Technical success	Completed, follow- up until 2027
	ENCIRCLE trial—NCT04153292	Prospective single-arm, multicenter study. Three cohorts: - main cohort, - failed TEER cohort, - MAC cohort	- MR ≥ 3+ - NYHA ≥ 2 - Ineligible to TEER - Inoperable	TS	n = 500	Composite of death and heart failure rehospitalization at 1 year.	February 2024
Evoque	MISCEND trial—NCT02718001	Single-arm, multicenter study	- Symptomatic MR - High surgical risk but operable	TS	n = 123	Major adverse events at 30 days	December 2023
Cephea	Cephea Early Feasibility Study—NCT05061004	Single-arm, multicenter study	- MR ≥ 3 - LVEF ≥ 30%	TS	n = 30	Safety: All-cause mortality at 30 days Efficacy: MR ≤ 2 at 30 days	June 2024
HighLife	HighFLO study NCT04888247	Single-arm, multicenter study in patients with high risk of LVOT obstruction	- MR ≥ 3+ - NYHA ≥ 2 - High surgical risk - High risk of LVOT obstruction	TS	n = 15	Technical success	December 2023
	Safety and Efficacy of the HighLife Transcatheter Mitral Valve Replacement in China—NCT05610566	Single-arm, multicenter study	- MR ≥ 3+ - NYHA ≥ 2	TS	n = 110	All-cause mortality at 12 months	July 2025
	Expanded Study of the HighLife 28 mm Trans-septal Trans-catheter Mitral Valve—NCT04029363	Single-arm, multicenter study	- MR ≥ 3+ - NYHA ≥ 2 - High surgical risk	TS	n = 120	Freedom from major adverse events at 30 days	January 2024

LVEF, left ventricular ejection fraction; MAC, mitral annular calcification; MR, mitral regurgitation; NYHA, New York Heart Association; TA, transapical; TEER, transcatheter edge-to-edge repair; TMVR, transcatheter mitral valve replacement, TS, transeptal.

## Data Availability

Data available in each original articles.
